# Observations and Modelling of the Pre-flare Period of the 29 March 2014 X1 Flare

**DOI:** 10.1007/s11207-017-1064-9

**Published:** 2017-02-14

**Authors:** M. M. Woods, L. K. Harra, S. A. Matthews, D. H. Mackay, S. Dacie, D. M. Long

**Affiliations:** 1 Mullard Space Science Laboratory, University College London, Holmbury St. Mary, Dorking, Surrey RH5 6NT UK; 2 School of Mathematics and Statistics, University of St Andrews, North Haugh, St Andrews, Fife FY16 9SS UK

**Keywords:** Flares, Pre-flare phenomena, Magnetic fields, Models

## Abstract

On 29 March 2014, NOAA Active Region (AR) 12017 produced an X1 flare that was simultaneously observed by an unprecedented number of observatories. We have investigated the pre-flare period of this flare from 14:00 UT until 19:00 UT using joint observations made by the *Interface Region Imaging Spectrometer* (IRIS) and the *Hinode Extreme Ultraviolet Imaging Spectrometer* (EIS). Spectral lines providing coverage of the solar atmosphere from the chromosphere to the corona were analysed to investigate pre-flare activity within the AR. The results of the investigation have revealed evidence of strongly blue-shifted plasma flows, with velocities up to $200~\mbox{km}\,\mbox{s}^{-1}$, being observed 40 minutes prior to flaring. These flows are located along the filament present in the active region and are both spatially discrete and transient. In order to constrain the possible explanations for this activity, we undertake non-potential magnetic field modelling of the active region. This modelling indicates the existence of a weakly twisted flux rope along the polarity inversion line in the region where a filament and the strong pre-flare flows are observed. We then discuss how these observations relate to the current models of flare triggering. We conclude that the most likely drivers of the observed activity are internal reconnection in the flux rope, early onset of the flare reconnection, or tether-cutting reconnection along the filament.

## Introduction

The dynamic magnetic environment of the solar atmosphere can lead to the storage of a large quantity of magnetic energy. This energy can be released via waves or magnetic reconnection, where the rapid energy release and its observational effects are manifested as a solar flare. Models of solar flare occurrence, such as the 2D CSHKP model (Carmichael, 1964; Sturrock, 1966; Hirayama, 1974; Kopp and Pneuman, 1976) and the recent 3D model (Janvier *et al.*, 2014), describe the flaring process and associated physical effects, but make little or no mention of the trigger mechanism. Recent work has identified several mechanisms and observational effects that could signify the flare trigger, but as yet, a definitive trigger model has not been forthcoming.

Prior to flare onset,“free” magnetic energy must be built up in the active region. This energy build-up can occur in a number of ways. Heyvaerts, Priest, and Rust (1977) suggested that the emergence of magnetic flux from below the solar surface could introduce new magnetic energy into an active region. This newly emerged flux can then interact with existing magnetic field in the solar atmosphere, resulting in the triggering of solar flares (see Priest, 2014 and references therein). Observational evidence for flux emergence comes through the use of magnetograms (both line-of-sight and vector). Solar flares are predominantly observed to occur at sites of strong magnetic field gradients. At these polarity inversion lines (PILs), energy can be built up in the field lines linking the two polarity regions through a shearing process. Shearing can be brought about by the rotation of sunspots (*e.g.*, Sundara Raman, Selvendran, and Thiagarajan, 1998), which can drag one polarity along the point of contact with the opposite polarity region. As rotation continues, shear increases, leading to a build-up of magnetic energy and eventually to reconnection resulting in a solar flare.

Once energy has built up in the system, several scenarios have been proposed for the initiation of large-scale energy release. Tether-cutting reconnection (Moore and Labonte, 1980; Moore *et al.*, 2001) is one such model of the initiation of flare and coronal mass ejection (CME) activity. This model proposes that slow reconnection can occur at the foot-points of a sheared loop system, resulting in the weakening of the overlying magnetic field. This weakening of the overlying field allows the filament that is supported by the sheared loop system to rise, in what is called the slow-rise phase. At some point during the rise, the system becomes torus unstable, and at the same time, a current sheet forms beneath the rising structure. Reconnection occurs in the current sheet, accelerating the filament in the fast-rise phase and causing flaring. This relationship between tether-cutting reconnection and the slow- and fast-rise phases of an eruption are supported by observational studies such as those by Chifor *et al.* (2006, 2007). In contrast to tether-cutting, Antiochos, DeVore, and Klimchuk (1999) presented the breakout model of flare or CME triggering. This model involves reconnection occurring above a sheared arcade at a magnetic null-point created between the arcade and overlying field. This reconnection weakens the overlying field and allows the eruption of magnetic flux from the centre of the arcade. Intensity enhancements during the pre-flare period such as those observed by Warren and Warshall (2001) away from the site of flaring have been linked to the breakout model.

The activation of the filament in an active region has also been linked to the occurrence of flaring. Rubio da Costa *et al.* (2012) discussed how small-scale reconnection within a filament leads to its destabilisation and subsequent flaring and eruption. Recent 3D magnetohydrodynamic (MHD) simulation work by Kusano *et al.* (2012) has identified small magnetic disturbances occurring near the PIL that may be a candidate for a flare trigger. A follow-up study by Bamba *et al.* (2013) investigated these disturbances and the small-scale internal reconnection that they cause through observation of Ca ii h emission line intensity enhancements.

Plasma instabilities are another possible way in which flaring can be triggered. Török and Kliem (2005) used MHD simulations to propose a model of solar eruptions triggered by the helical kink instability. This instability is triggered when twist in a magnetic flux rope (MFR) exceeds a critical value and causes the MFR to kink and rise upwards. There have been observations that support this model, such as Williams *et al.* (2009), who observed asymmetric Doppler shifts along a filament prior to eruption, which they interpreted as an MFR subjected to the effects of the kink instability. Kliem and Török (2006) proposed a further plasma instability as a trigger mechanism, the torus instability. In this model, if the Lorentz force provided by an external magnetic field decreases faster than the hoop force exerted by an expanding current ring embedded in the external field, the system will be unstable. These instabilities can then rapidly cause the current ring to rise, triggering an eruption. Authors such as Zuccarello *et al.* (2014) have provided observational evidence for the role of torus instabilities in triggering eruptions.

The trigger mechanisms discussed thus far have clearly defined observational signatures. Pre-flare features have been identified, however, that are not yet associated with a specific flare trigger model. Non-thermal velocity ($V_{\mathrm{nt}}$) enhancements have been identified up to an hour prior to flares in X-rays by Doschek *et al.* (1980), and tens of minutes prior by Harra, Matthews, and Culhane (2001). In EUV wavelengths Harra *et al.* (2009) and Wallace *et al.* (2010) observed $V_{\mathrm{nt}}$ enhancements over an hour before flare occurrence, and Harra *et al.* (2013) related this type of enhancement to sites of coronal dimming. They also suggested that these enhancements could be another indicator of filament activation prior to flaring.

The X-class flare of 29 March 2014 has been a source of intense study by many authors, due to the quality and variety of data available (*e.g.* Judge *et al.*, 2014; Matthews *et al.*, 2015; Li *et al.*, 2015; Battaglia *et al.*, 2015; Young, Tian, and Jaeggli, 2015; Liu *et al.*, 2015; Aschwanden, 2015; Kleint *et al.*, 2016; Rubio da Costa *et al.*, 2016). There have been studies investigating aspects of the pre-flare period of this flare. Abramov-Maximov *et al.* (2015) carried out microwave and radio observations of the active region that produced the X-flare, following the evolution of the region from its appearance on disk until flaring. Yang, Guo, and Ding (2016) used vector magnetic field extrapolations to investigate the magnetic field configuration in the active region between 28 and 29 March 2014. This work identified the presence of a magnetic flux rope in this region and suggested that the majority of the flares during their period of study were triggered by the kink instability. Kleint *et al.* (2015) studied the filament eruption of this flare in detail, finding that the filament exhibited accelerations of ${\approx}\,3\,\mbox{--}\,5~\mbox{km}\,\mbox{s}^{{-2}}$ during the eruption. They also identified small Doppler shifts (velocities of $2\,\mbox{--}\,4~\mbox{km}\,\mbox{s}^{-1}$) along the filament up to an hour prior to the flare observed by the *Interferometric BIdimensional Spectrometer* (IBIS), which they attributed to either plasma flows or the slow rise of the filament.

Whilst these previous studies investigated aspects of the pre-flare behaviour of this flare, none carried out a dedicated spectroscopic investigation of pre-flare activity. We therefore present the first spectroscopic study of pre-flare activity to simultaneously observe the chromosphere, transition region, and corona using data from *Hinode*/EIS and IRIS spectrometers. Section 2 provides an overview of the observations. We present the findings of these observations in Section 3, including the identification of transient strongly blue-shifted features that are characterised by transition region velocities of up to $200~\mbox{km}\,\mbox{s}^{-1}$ measured in emission line wings. These features are determined to be plasma flows and seen up to ${\approx}\,40~\mbox{minutes}$ prior to flare onset. We discuss how these features relate to extended bright features observed alongside the filament in the run up to the flare. The results of non-potential magnetic field modelling of the active region are presented, revealing the presence of a magnetic flux rope. Section 4 contains discussion of the results and how they may relate to current models of flare triggering.

## Observations

**Figure 1:**
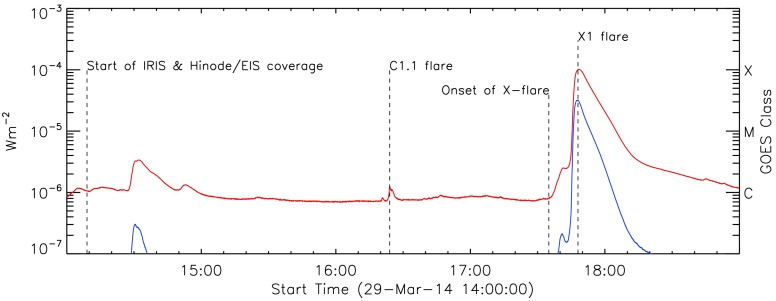
GOES light curve of the soft X-ray flux from 29 March 2014 14:00 UT. Joint *Hinode*/EIS and IRIS coverage starts at 14:09 UT. The flare at 14:30 UT occurs outside the spectrometer field of view. At 16:24 UT a C1.1 flare was observed by both spectrometers, followed by the X1 flare at the peak time of 17:48 UT.

Beginning at SOL2014-03-29T17:35, *National Oceanographic and Atmospheric Administration* (NOAA) Active Region (AR) 12017 produced an X1 flare. This flare was observed by an unprecedented number of observatories, both in space and on the ground. Figure 1 shows the GOES soft X-ray light curve from 14:00 UT, with the times of relevant events marked.

**Figure 2:**
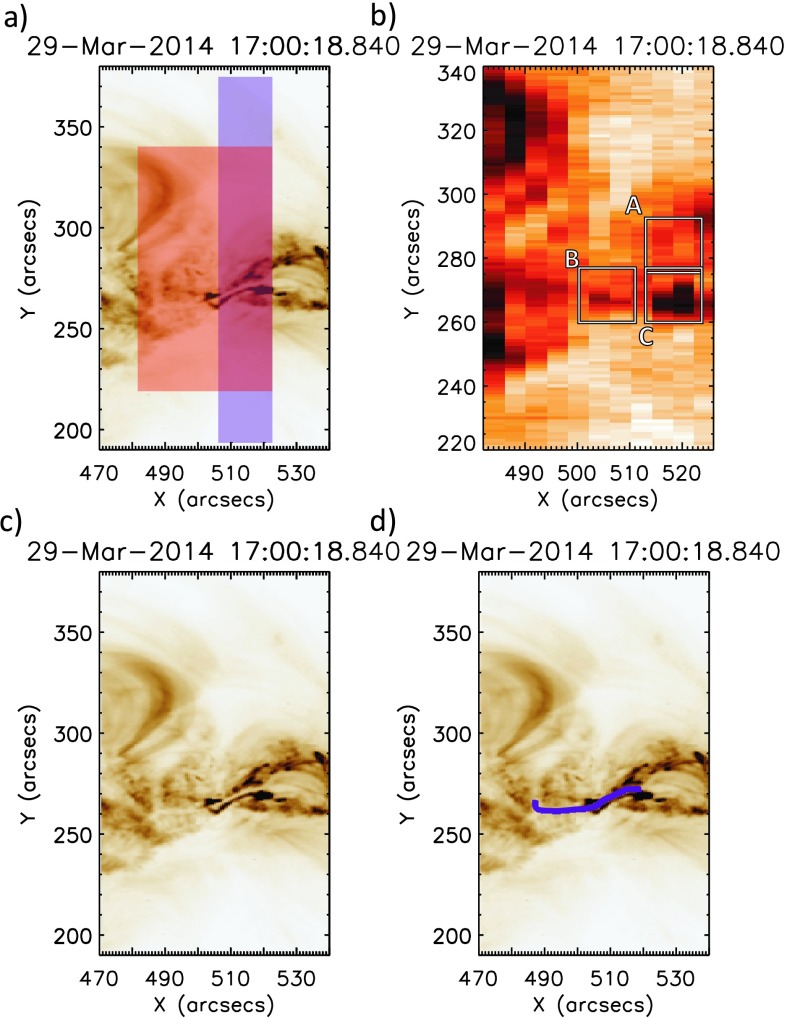
Panel (a) shows the fields of view of the IRIS (blue) and *Hinode*/EIS (red) spectrometers overlaid onto the active region seen in the 193 Å AIA channel, presented with an inverted colour table. All images have been differentially rotated to the time of the closest AIA 193 Å exposure at the time of the central *Hinode*/EIS raster step. Panel (b) shows the three regions of study and their positions within the *Hinode*/EIS Fe xii field of view. The position of the filament is clearly seen in the 193 Å and 304 Å AIA channels, with panel (c) showing the 193 Å data. The path of the filament is marked in panel (d) by the purple line for emphasis.

The *Extreme Ultraviolet Imaging Spectrometer* (EIS; Culhane *et al.*, 2007) onboard the *Hinode* spacecraft (Kosugi *et al.*, 2007) was observing AR 12017 continuously from 14:05 – 17:57 UT. During this time, 104 rasters of the field of view were produced with a cadence of 134 s. The observing program makes use of the $2''$ slit rastering across a field of view of $42'' \times 120''$ in $4''$ steps (shown in red in Figure 2, panel (a)). Eight spectral windows are contained within the observing program, of which Fe xii 192.39 Å and He ii 256.28 Å are selected for analysis. Fe xii is selected as it is the strongest coronal line observed by EIS providing the best opportunity of identifying low-intensity pre-flare activity. He ii was chosen to study the lower atmosphere because of its pseudo-chromospheric nature.

The *Interface Region Imaging Spectrometer* (IRIS; De Pontieu *et al.*, 2014) was observing AR 12017 from 14:09 – 17:54 UT with a field of view of $14'' \times 174''$, produced using an eight-step raster of $2''$ steps (highlighted in blue in Figure 2, panel (a)). Although nominal exposures were 8 seconds, automatic exposure control was in effect during rasters covering the peak of the X-flare, reducing the exposure time to ${\approx}\,2$ seconds. These exposure times result in a raster cadence of 72 s. IRIS also uses a slit-jaw imager (SJI) to provide context to spectroscopic observations.

Data from NASA’s *Solar Dynamics Observatory* (SDO; Pesnell, Thompson, and Chamberlin, 2012) *Atmospheric Imaging Assembly* (AIA) and *Helioseismic and Magnetic Imager* (HMI) instruments were downloaded with calibrations and corrections already applied. The routine aia_prep was run on the data before use to account for small scaling differences between the full-disk images produced by both instruments.

In order to directly compare spatial locations between the four data sets used in this analysis, *Hinode*/EIS and IRIS images are aligned with AIA images. This alignment is done manually using feature recognition of the corresponding AIA wavelength to the chosen *Hinode*/EIS or IRIS data. To do this, *Hinode*/EIS Fe xii 192.6 Å data are aligned to AIA 193 Å data for each time step, while for the IRIS alignment, Si iv 1400 Å observations are aligned to the AIA 1600 Å channel. The alignment by feature recognition requires only very small changes to position, of no more than ${\pm}\,2~\mbox{arcseconds}$ for each individual raster.

## Results

### Active Region Evolution

**Figure 3:**
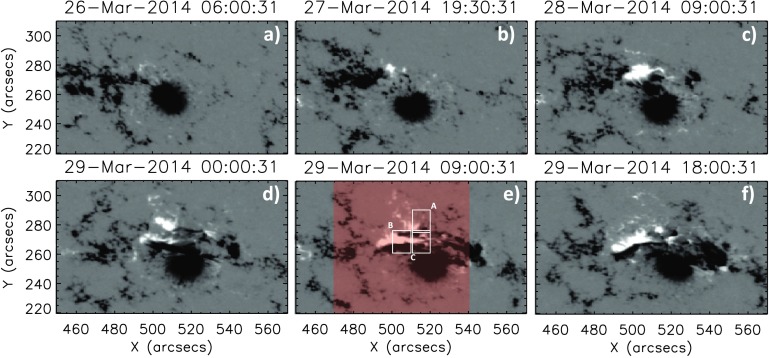
Evolution of the central portion of AR 12017 between 26 March 2014 and 29 March 2014. Flux emergence is observed in panel (b), and subsequent panels show the formation of a highly sheared polarity inversion line. Plots are scaled between ${\pm}\,550~\mbox{Gauss}$. Panel (e) also indicates the AIA field of view used in Figure 2, marked by the red shaded area, as well as the three regions of study.

Active Region 12017 was first observed crossing the eastern limb of the Sun on 22 March 2014. The evolution of this active region from 22 March 2014 to 29 March 2014 was discussed in Abramov-Maximov *et al.* (2015). They showed that there is a large negative-polarity sunspot leading the active region with smaller less coherent positive polarity trailing until 27 March 2014, when a new positive-polarity region emerges next to the leading negative polarity. This is illustrated in Figure 3, which shows the evolution of the central portion of the active region. Panel (a) displays the initial morphology, with the clear large negative sunspot. In panel (b), we observe the emergence of the positive-polarity region. Panels (c) – (f) show the evolution of this emergence and its subsequent interaction with the existing negative polarity. It is interesting to note that as this interaction progresses, the apparent motion of the positive polarity with respect to the polarity inversion line (PIL) indicates an increase in shear of the magnetic field. Panel (f) shows the active region just after the peak of the X-class flare. We see that the field has become sheared over the PIL, suggesting that there has been a large build-up of magnetic energy in this region.

Within the active region on 29 March 2014, there is a clearly visible filament. This feature can be seen clearly in the AIA 193 Å observations in Figure 2, panels (c) and (d). The path of the filament can also be clearly seen in the $\mbox{H}\upalpha$ observations presented in Figure 5 of Kleint *et al.* (2015).

### Pre-flare Observations in the Corona

Previous studies of pre-flare behaviour have found enhanced $V_{\mathrm{nt}}$ features observed in the corona over periods of tens of minutes to an hour prior to flare onset, suggesting increased $V_{\mathrm{nt}}$ may be an indicator of imminent flare onset (Harra *et al.*, 2009). In order to investigate whether this phenomenon was observed prior to the 29 March 2014 X-flare, Fe xii 192.3 Å observations from *Hinode*/EIS are analysed. These data are fitted with single Gaussian profiles. Owing to the lack of absolute wavelength calibration in *Hinode*/EIS data, care is taken to determine a rest wavelength. This is done by selecting a small area of the field of view from a pre-flare raster in which little activity is observed and fitting to determine the rest wavelength. The raster chosen for this purpose is the first in the data set, recorded at 15:01:09 UT. Values for the Doppler velocity and $V_{\mathrm{nt}}$ are determined from the results of the spectral fitting. The Doppler velocity is determined by measuring the deviation of line centre from the rest velocity, while $V_{\mathrm{nt}}$ is defined as the width of a line observed above that of the theoretical thermal line width for the given ion under observation.

**Figure 4:**
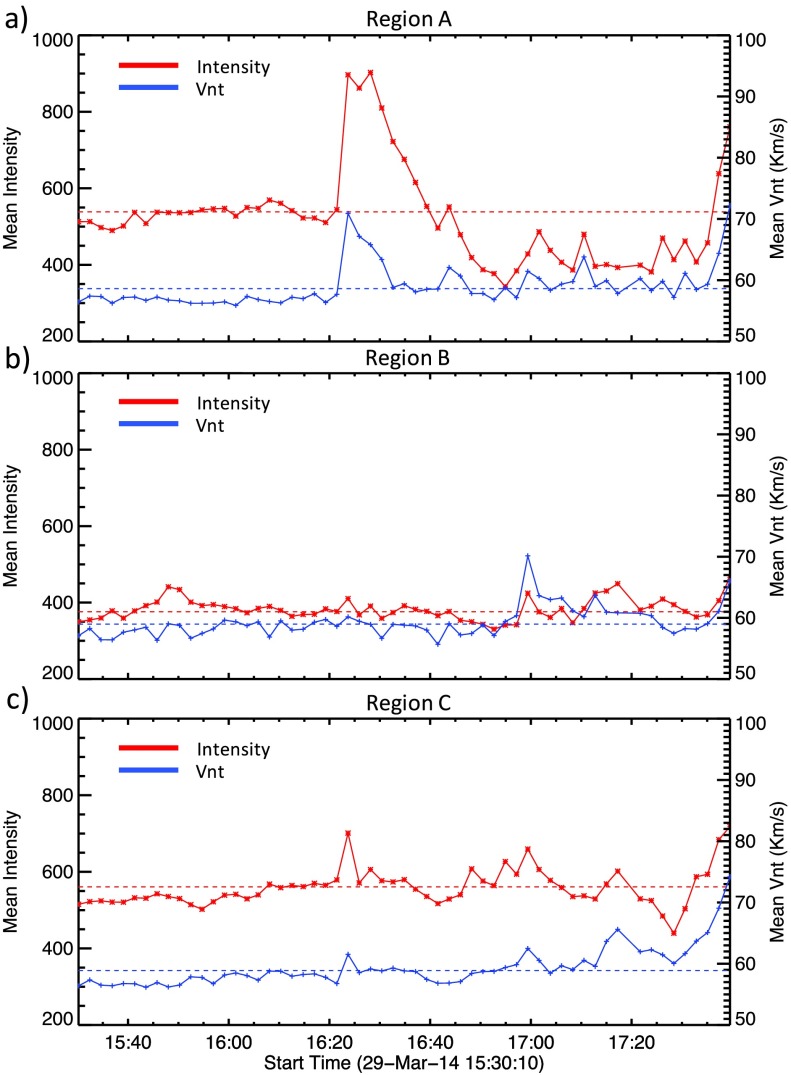
Evolution of mean intensity (red) and mean $V_{\mathrm{nt}}$ (blue) calculated for Fe xii in time for Regions A, B, and C, as shown in Figure 2. In Region A, shown in panel (a), the two profiles broadly match, in particular showing a clear peak during the 16:24 UT C1.1 flare. The location of $V_{\mathrm{nt}}$ enhancement during the C-flare is shown in Figure 5 panel (a). In Region B, panel (b), we can see that at 17:00 UT, non-thermal velocity peaks at a value of ${\approx}\,70~\mbox{km}\,\mbox{s}^{-1}$. This non-thermal velocity peak occurs in the absence of a corresponding intensity increase. The location of this region of $V_{\mathrm{nt}}$ enhancement is shown in Figure 5 panel (b). Region C, panel (c): both intensity and non-thermal velocity profiles match well, with no irregularities as in the case of Region B. A smaller response to the C1.1 flare is observed in this region. Prior to 17:00 UT, increases in intensity and non-thermal velocity are observed. Region C also exhibits the earliest response to the X flare of the three regions studied. Figure 5 panel (c) shows the location of non-thermal velocity enhancements.

**Figure 5:**
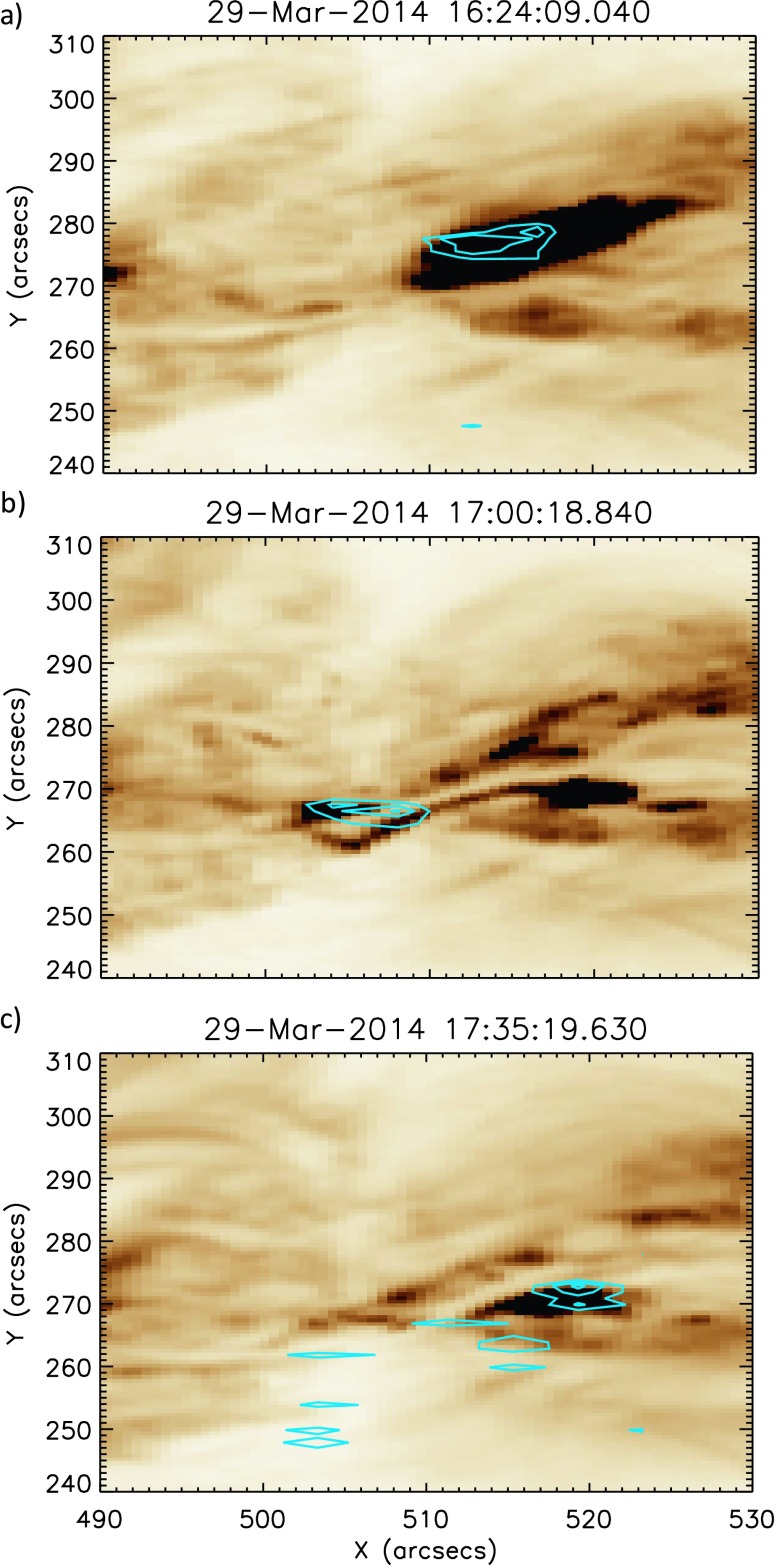
This figure shows non-thermal velocity contours overlaid onto corresponding AIA 193 Å images to exhibit the location of the non-thermal velocity enhancements. $V_{\mathrm{nt}}$ contours are plotted between 70 and $200~\mbox{km}\,\mbox{s}^{-1}$, and the AIA colour scale is inverted to improve clarity. Panel (a) shows the location of these enhancements during the C-flare at 16:24 UT. Panel (b) shows that the $V_{\mathrm{nt}}$ enhancements seen at 17:00 UT are located in a region in the centre of the filament. Enhancements seen at the start on the X-flare, at 17:35 UT, are shown in panel (c).

Figure 4 shows time profiles of mean intensity (red) and mean $V_{\mathrm{nt}}$ (blue) for three chosen subregions: A, B, and C. This method of investigating the behaviour using $V_{\mathrm{nt}}$ averaged over a small region is similar to that employed in earlier coronal studies of pre-flare activity (*e.g.* Harra *et al.*, 2009, 2013). Region A is chosen as it is the site of the confined C-class flare that occurred at 16:24 UT. From Figure 4 panel (a), we see that the intensity and $V_{\mathrm{nt}}$ time profiles match closely, both exhibiting a strong peak during the C-class flare. This region of $V_{\mathrm{nt}}$ enhancement is clearly seen in panel (a) of Figure 5, where Fe xii $V_{\mathrm{nt}}$ contours are overlaid onto AIA 193 Å images at the peak of the C-flare. The time profile of Region B (Figure 4, panel (b)) shows little activity early in the observations as this region is uninvolved in the C-flare at 16:24 UT. However, at ${\approx}\,17\mbox{:}00~\mbox{UT}$ there is a clear spike in the mean $V_{\mathrm{nt}}$ for Region B, accompanied by a small increase above the mean intensity. The peak mean $V_{\mathrm{nt}}$ observed in Region B is over $70~\mbox{km}\,\mbox{s}^{-1}$. This is a significant value for an active region at a time when no flaring activity is occurring. Testa, De Pontieu, and Hansteen (2016) have found $V_{\mathrm{nt}}$ values in non-flaring active regions to be $24~\mbox{km}\,\mbox{s}^{-1}$, as determined from the Fe xii 1349.4 Å emission line. It can be clearly seen in panel (b) of Figure 5 that this small section of $V_{\mathrm{nt}}$ enhancement is located directly above the area of the active region where the filament is situated. From the Doppler velocity data, this feature is identified as having a line-centre blue shift of tens of $\mbox{km}\,\mbox{s}^{-1}$. The time profile of Region C (Figure 4, panel (c)) is again different to that seen in the other regions. At 16:24 UT, there is a small response to the C 1.1 flare seen in both intensity and $V_{\mathrm{nt}}$. This weaker response to the C-flare in this region results from the effects of the flare only being present in a small subregion that is common to both Regions A and C. Region C also shows intriguing activity from ${\approx}\,16\mbox{:}45~\mbox{UT}$ to 17:00 UT, both in intensity and $V_{\mathrm{nt}}$. The intensity profile during this period exhibits three periodic peaks, each with a higher peak intensity than the previous peak. This periodic increase in intensity is accompanied by a general increase in $V_{\mathrm{nt}}$. At ${\approx}\,17\mbox{:}17~\mbox{UT}$, there is a peak in intensity and $V_{\mathrm{nt}}$. The onset of the X-class flare is seen in Region C from ${\approx}\,17\mbox{:}28~\mbox{UT}$ with clear and sustained increases in intensity and $V_{\mathrm{nt}}$. These effects of the X-flare are seen in Region C up to five minutes prior to similar effects in Regions A and B. $V_{\mathrm{nt}}$ enhancements at the start of the X-flare (17:35 UT) can be seen in panel (c) of Figure 5. Having found sources of enhanced coronal $V_{\mathrm{nt}}$, we explore the behaviour of the lower atmosphere at the pixel scale.

### Response of the Lower Atmosphere

The response of the lower atmosphere in these regions is also investigated to fully understand pre-flare activity throughout the solar atmosphere. The transition region response is investigated via the Si iv line at 1402.77 Å observed by IRIS. The pseudo-chromospheric He ii 256.2 Å line observed by EIS and the optically thick chromospheric Mg ii h and k lines (at 2803.52 Å and 2796.34 Å, respectively) observed by IRIS are chosen to investigate the atmosphere to chromospheric depths. To investigate the dynamics in these regions, the evolution of the profile of each spectral line is studied between 16:16 UT and 18:00 UT. The data for each line profile sequence are plotted as a function of time and Doppler velocity, centred on the rest velocity of each individual line. These rest velocities are calculated by fitting a small inactive area of a pre-flare raster. The following is an account of the pre-flare activity observed for each sub-region in turn.

#### Region A: Site of C-class Flare

**Figure 6:**
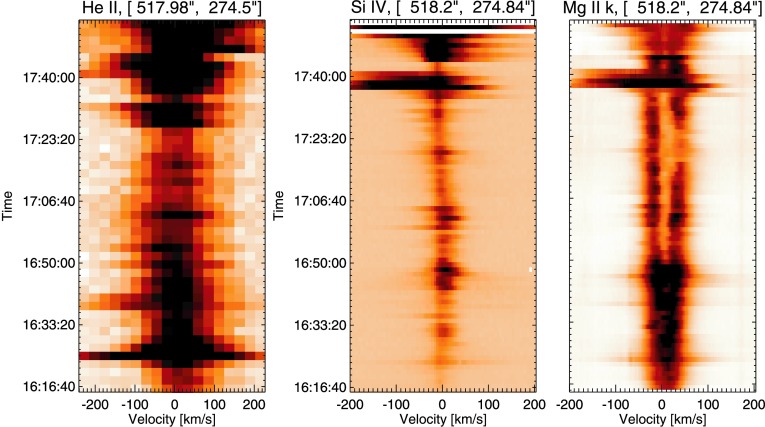
Spectral time profiles recorded in Region A at 16:16 UT. Panel (a) shows He ii spectra, panel (b) shows Si iv spectra, and panel (c) shows the evolution of the Mg ii k line during this time period. Broadening of the lines is observed at 16:24 UT in response to the C-flare. The response to the X-class flare can be seen in all lines from ${\approx}\,17\mbox{:}35~\mbox{UT}$.

In Region A (Figure 4, panel (a)), the intensity and $V_{\mathrm{nt}}$ profiles track each other closely, both exhibiting a strong peak during the C1.1 flare at 16:24 UT. Figure 6 illustrates the He ii, Si iv, and Mg ii k line profile evolution for Region A. We clearly see in the He ii spectra the increase in intensity and line width that is due to the C1.1 flare at 16:24 UT, as well as weaker dynamics in both lines up until the onset of the X-flare at 17:35. Both He ii and Si iv spectra show a strongly blue-shifted feature at ${\approx}\,17\mbox{:}40~\mbox{UT}$, which we interpret as the eruption of the filament during the X-flare. The Mg ii k emission is observed to have strong intensity and a weak central reversal in its profile from the onset of the C-flare until ${\approx}\,16\mbox{:}50~\mbox{UT}$. At this point in time, the central reversal deepens and the line intensity is weaker on the whole. As in the case of He ii and Si iv, strong blue asymmetries are seen in the Mg ii data at ${\approx}\,17\mbox{:}40~\mbox{UT}$. Thus within Region A, we observe a similar response to the C-flare throughout the atmosphere, noting that the activity is in line with that expected for an area of study such as this.

#### Region B: Site of Pre-flare $V_{\mathrm{nt}}$ Enhancement

**Figure 7:**
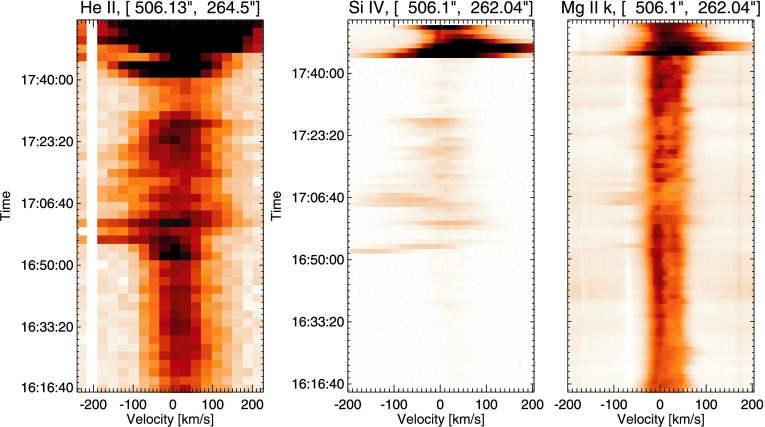
Spectral time profiles recorded in Region B at 16:16 UT. Panel (a) shows He ii spectra, panel (b) shows Si iv spectra, and panel (c) shows the evolution of the Mg ii k line during this time period. Little activity is observed until the onset of strong blue shifts is seen from ${\approx}\,16\mbox{:}52~\mbox{UT}$. The onset of the flare can be seen to occur in this region from 16:40 UT.

Region B is chosen for further study because of an intriguing $V_{\mathrm{nt}}$ feature observed at 17:00 UT. Figure 7 shows the evolution of the He ii, Si iv, and Mg ii k line profiles in Region B. As expected, there is no observed activity during the C1.1 flare as this is confined solely to Region A. From the start of observation until ${\approx}\,16\mbox{:}50~\mbox{UT}$, the spectra show ordinary active region dynamics with little to no line broadening or Doppler shifts. From ${\approx}\,16\mbox{:}50~\mbox{UT}$, however, strong blue shifts of up to $200~\mbox{km}\,\mbox{s}^{-1}$ are observed to initiate very rapidly in both He ii and Si iv lines. Additionally, these strong blue shifts are observed to be very dynamic, reaching a maximum around 16:54 UT, falling at 17:00 UT, and then reaching $200~\mbox{km}\,\mbox{s}^{-1}$ from 17:04 UT. This behaviour is very interesting as peak coronal blue shifts, determined from Fe xii data, are observed at 17:00 UT, suggesting there is a temporal offset between the layers of the atmosphere involved in the activity. After these peak blue shifts, the line profiles continue to be dynamic until the onset of the X-flare, albeit exhibiting blue shifts of lower velocities of up to ${\approx}\,100~\mbox{km}\,\mbox{s}^{-1}$. Within this region, we identify increased $V_{\mathrm{nt}}$ from coronal Fe xii data, as well as strong blue-shifted flows in the lower atmosphere that initiate very rapidly.

#### Region C: Site of Pre-flare Brightening and Earliest Response to X-Flare

**Figure 8:**
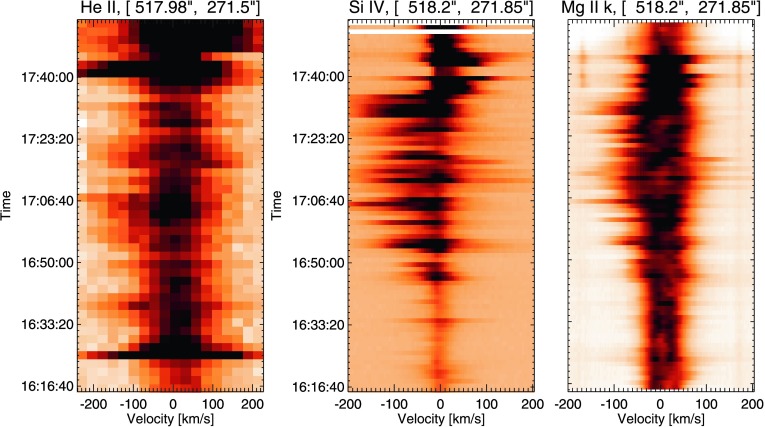
Spectral time profiles recorded in Region C at 16:16 UT. Panel (a) shows He ii spectra, panel (b) shows Si iv spectra, and panel (c) shows the evolution of the Mg ii k line during this time period. A small response to the 16:24 UT C-flare is observed in this region. From 16:45 UT until the onset of the X-flare, blue-shifted line broadening is observed.

Region C is chosen as it is the site of a pre-flare brightening observed in AIA 193 Å data. The single-pixel time profiles obtained in Region C, Figure 8, differ again from those observed in Regions A and B. Little activity or line broadening is observed in Si iv and Mg ii k spectra prior to ${\approx}\,16\mbox{:}45~\mbox{UT}$. The He ii time profile shows a response to the C-flare at 16:24 UT and in general shows more activity than the other lines studied during this time period. From 16:45 UT until the onset of the X-class flare at 17:35 UT, this activity is characterised by increased line intensity, especially in Si iv observations, as well as strong intermittent blue asymmetries in all observed lines. These blue asymmetries have speeds between $100~\mbox{km}\,\mbox{s}^{-1}$ and $200~\mbox{km}\,\mbox{s}^{-1}$. The onset of pre-flare activity in this region is not as dramatic as that in Region B, but dynamic blue shifts of up to $200~\mbox{km}\,\mbox{s}^{-1}$ are observed.

### Location of Observed Plasma Flows

**Figure 9:**
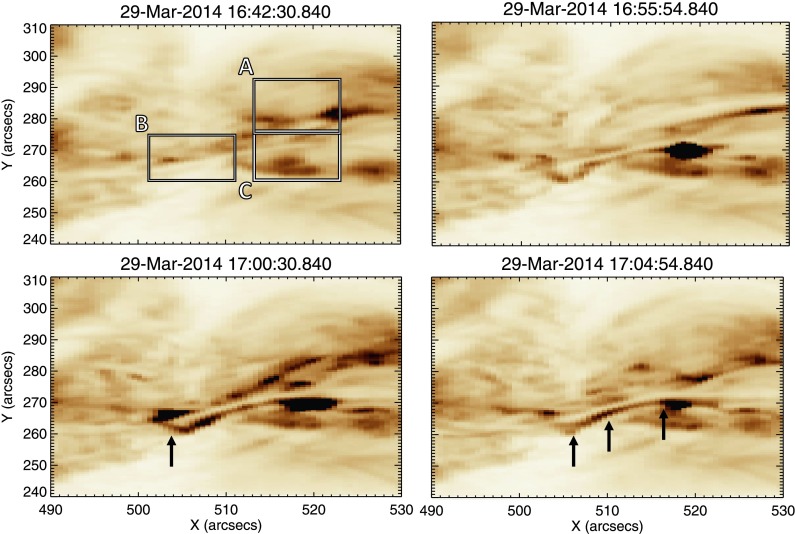
Stills detailing coronal activity observed by SDO/AIA in 193 Å, shown using an inverted colour table, between 16:42 UT and 17:04 UT. Panel (a) shows the filament prior to any observed activity. Overlaid are the three sub-regions of study. Panel (b) shows the appearance of a bright feature to the west of the filament. Panel (c) shows a loop feature crossing the filament at the site of the peak coronal blue shift as observed by *Hinode*/EIS. This activity is marked by the black arrow in the diagram. Panel (d) shows an extended bright feature lying along the southern edge of filament. The path of this brightening is marked by the overlaid black arrows.

**Figure 10:**
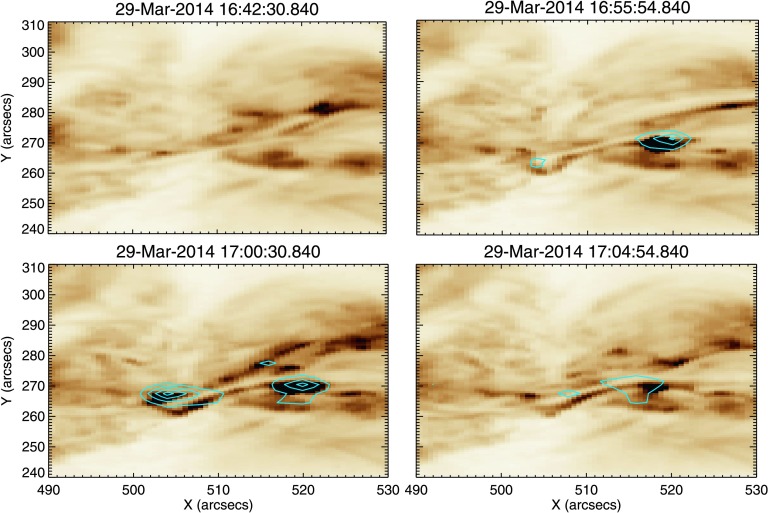
Here we see the same AIA 193 Å (colour table inverted) field of view as Figure 9, over-plotted with *Hinode*/EIS Fe xii data at $-100~\mbox{km}\,\mbox{s}^{-1}$. No strong blue shifts can be seen in the EIS data in panel (a) at 16:42 UT. By 16:55 UT (panel (b)), we can see that there is a strong area of blue shift located over a bright region in the AIA data. There is also a less intense region of blue shift located in the centre of the filament. At 17:00 UT (panel (c)), both these blue-shifted regions have increased in intensity. Panel (b) shows the situation at 17:04 UT, where the strong blue shifts have dissipated greatly with the feature in the centre of the filament being no longer visible.

**Figure 11:**
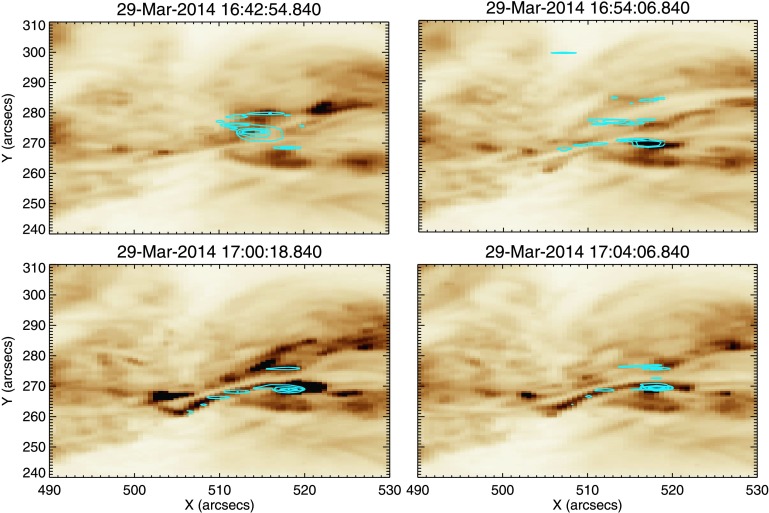
For the same region detailed in Figures 8 and 9, this figure displays Si iv emission at $-100~\mbox{km}\,\mbox{s}^{-1}$ observed by IRIS, overlaid onto AIA 193 Å data (colour table inverted). In panel (a), at 16:42 UT, we see a blue-shifted region centred on the region of the earlier C-class flare. In panel (b), 16:54 UT, blue shifts in the region of the C-flare have waned in intensity. In the region of brightening in the AIA data there is a region of strong blue shift, as well as discrete areas of blue shift extending along the filament. At 17:00 UT, panel (c), these discrete blue shift features are located along the extended bright feature visible in AIA data. By 17:04 UT, panel (d), blue shifts have decreased in intensity, but are still present along the extended bright feature.

From examination of the line profiles we determine that from ${\approx}\,16\mbox{:}50~\mbox{UT}$ the plasma is highly dynamic. As these line profiles represent the evolution of the spectra at one pixel in time, we must also consider the morphology of these flows and their evolution over the whole field of view. Figure 9 shows four AIA 193 Å images charting the activity along the filament. In panel (a) we see the filament at 16:42 UT and note that there is little activity seen in the areas corresponding to Regions A, B, and C (positions are shown in the marked boxes). Panel (b) shows a bright feature appearing within Region C, lying directly to the south of the filament, at around 16:54 UT. By 17:00 UT (panel (c), the time of peak coronal blue shift as measured by *Hinode*/EIS), we can see that a bright loop now lies across the filament, within Region B. This loop is situated directly above the site of the blue shifts observed by *Hinode*/EIS and IRIS. Additionally, in both panels (c) and (d) an elongated bright feature extends from this loop westwards to the site of the initial brightening observed in panel (b). The path of this feature is marked by the black arrows in panel (d). In Figure 10 we show for similar time selections the Fe xii data at $-100~\mbox{km}\,\mbox{s}^{-1}$ overlaid onto the AIA data. We see from panel (a) that there is no activity at this velocity in the Fe xii data at 16:42 UT. An area of strong blue shift is seen to appear in the region of increased intensity at 16:54 UT, along with a weaker blue-shifted feature in the centre of the filament, shown in panel (b). Panel (c) shows that at 17:00 UT this blue-shifted region is still present, along with a second strongly blue-shifted region in the centre of the filament. Panel (d) of Figure 10 shows that at 17:04:54 UT the blue shifts in the corona seem to extend from the two regions identified earlier, along the extended bright feature. Figure 11 details the morphology of the $-100~\mbox{km}\,\mbox{s}^{-1}$ blue shifts observed in the Si iv data, overlaid onto the AIA 193 Å images. Panel (a) reveals that at 16:42 UT, blue shifts in the region of the earlier C-class flare occur. At 16:54 UT (panel (b)), blue shifts are still present at the site of the C-class flare, but to a lesser extent. In the region of the bright feature in the AIA 193 Å image, strong blue shifts are also observed. These blue shifts are in the same positions as those identified during the same time period from Fe xii data. Additional blue shifts are also observed along the filament at this point in time. In panel (c), we find that the blue shifts associated with the bright feature are still present. Blue shifts are also observed to lie along the bright ribbon-like feature to the south of the filament. It is noted that these blue shifts are spatially discrete. The situation seen in panel (d) at 17:04 UT is similar to that observed at 17:00 UT. However, the positions of the spatially discrete blue shifts along the extended bright feature have changed, highlighting the transient nature of these features. This behaviour is seen more clearly in the movie of this time period (Movie 1) in the online version.

The fast plasma flows and brightenings, observed throughout the solar atmosphere and discussed in the preceding sections, are clear examples of pre-flare activity. However, several differing models could be used to explain such activity. In order to attempt to confine the possible drivers, non-potential magnetic field modelling is carried out on the active region. This work and its results are described in Section 3.5.

### Non-potential Magnetic Field Modelling

To simulate the non-potential evolution of the active region magnetic field and the morphology of the magnetic field at the filament location, a continuous time-series of quasi-static, non-linear force-free fields are produced. These non-potential magnetic fields are produced using the technique developed and applied in Mackay, Green, and van Ballegooijen (2011) and Gibb *et al.* (2014). In this technique, the boundary driving at the level of the photosphere is obtained directly from a time series of HMI line-of-sight magnetograms, which are directly applied as lower boundary conditions. The coronal magnetic field then responds to these motions by evolving through a continuous series of quasi-static non-linear force-free fields using the magneto-frictional relaxation method. Full details of the equations solved and the technical details of applying this technique to magnetogram data are described in the cited articles.

**Figure 12:**
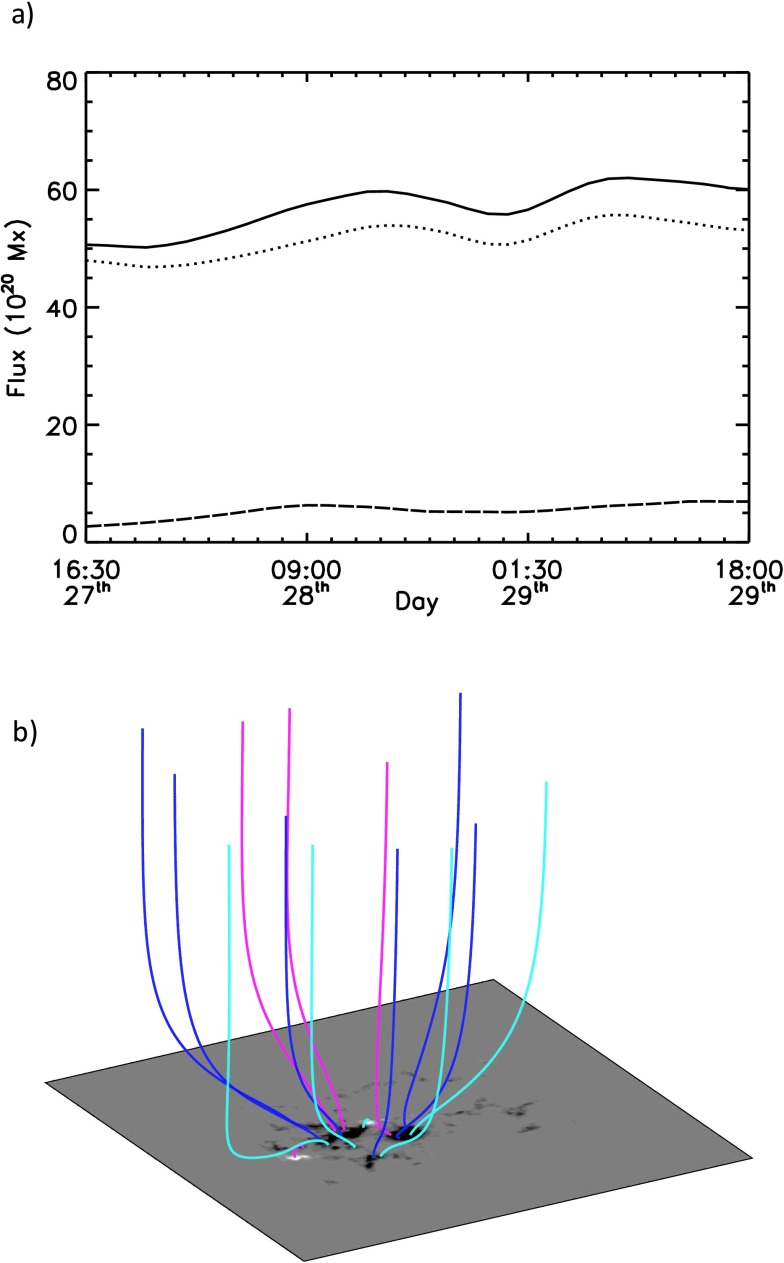
(a) Graph of the variation of total flux (solid line), positive flux (dashed line), and absolute value of negative flux (dotted line) over the time period of the simulation. (b) Initial potential field configuration used in the simulation corresponding to a start time of 16:30:31 UT on 27 March 2014.

The time period of the simulation ranges from 16:30:31 UT on 27 March 2014 to 19:30:31 UT on 29 March 2014, where the cadence of the magnetograms is taken to be 90 minutes. This time period is fully illustrated in Figure 3, where a selection of the magnetograms can be seen. From these panels it is clear that the area of interest is dominated by negative flux. While this is the case, from around 19:30:31 UT on 27 March 2014 a new positive polarity of a bipole begins to appear and then grows strongly over the next two days. The variation of the total flux (solid line), unsigned negative flux (dotted line), and positive flux (dashed line) are seen in Figure 12 panel (a). From this plot it can be seen that both the positive and negative flux increase over time, characterised by the emergence of new flux. The numerical simulations of this active region are carried out in a computational box of $512^{3}$ grid points. Closed-side boundary conditions are applied, but because of the dominance of negative flux, open-top boundary conditions are used. Use of the open-top boundary condition means that no correction for flux balancing is required at the modelled photosphere. For use in the model, the time series of full-disk magnetograms are de-rotated to disk centre and a portion of size $300 \times 210$ pixels is extracted and then centred at the lower boundary in the computational box. The normal field component on all computational grid points outside of the area on the magnetogram are set to zero. A potential magnetic field is then constructed from the initial magnetogram (Figure 12 panel (b)). Owing to the dominance of negative flux, approximately 90% of the flux is open.

**Figure 13:**
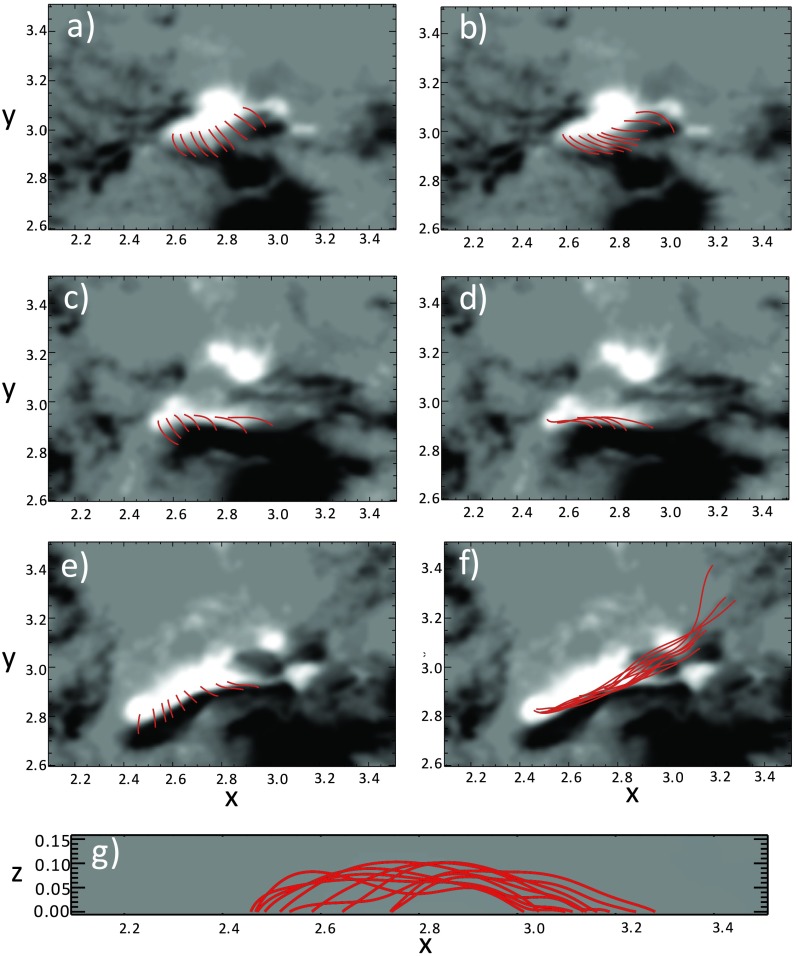
Connectivity of the field lines along the location of the filament at 09:30:31 UT on 28 March 2014, (a) and (b), at 00:00:31 UT on 29 March 2014, (c) and (d), and at 16:30:31 UT on 29 March 2014, (e), (f), and (g). Panels (a) to (f) show the field lines from above superimposed on the magnetogram (white represents positive flux, and black represents negative flux), while panel (g) shows the field lines from panel (f) viewed from the side. Finally, panels (a), (c), and (e) do not include the additional helicity injection term, while panels (b), (d), (f), and (g) include additional helicity injection at a rate of $3.75\,\mbox{--}\,5 \times10^{16}~\mbox{Mx}^{2}\,\mbox{cm}^{-2}\,\mbox{s}^{-1}$.

In Figure 13 panel (a) the field lines produced in the quasi-static non-linear force-free field simulation can be seen at 09:30:31 UT on 28 March 2014. In this plot, white represents positive flux and black negative flux. The field lines passing over the PIL are illustrated by the red lines. From this it is clear that these field lines do not exhibit a strong magnetic shear. Similar weak magnetic shear of the field is found in the corresponding field line connectivity at 00:00:31 UT on 29 March 2014 (Figure 13 panel (c)) and 16:30:31 UT on 29 March 2014 (Figure 13 panel (e)). Therefore none of these magnetic configurations produce a strongly sheared magnetic field along the PIL at the correct location or time that is representative of the field of a filament. From this we therefore find that for the present case the horizontal motions deduced from the magnetograms do not inject enough non-potentiality or helicity into the coronal field to produce a strongly sheared magnetic field at the observed position of the filament.

To investigate the possible origin of the strongly sheared non-potential magnetic structure of the filament, a second series of simulations are carried out where an additional injection of helicity is included at the photosphere. This is included through the addition of Equation (A1) from the article of Mackay, DeVore, and Antiochos (2014). Inclusion of this term allows for the injection of either positive or negative helicity at the photosphere without changing the normal magnetic field. Figure 13 panel (b) shows the field lines at 09:30:31 UT on 28 March 2014, when an average relative helicity density of $5 \times 10^{16}~\mbox{Mx}^{2}\,\mbox{cm}^{-2}\,\mbox{s}^{-1}$ is injected into the corona throughout the simulation. It is clear that with this additional helicity injection, the field lines now exhibit a strong shear that is directed to the left (sinistral) when standing on the positive polarity side of the PIL. This strong shear along the PIL continues to build, such that at 00:00:31 UT on 29 March 2014 (Figure 13 panel d), there is an arcade lying directly along the PIL that then evolves into a magnetic flux rope by 16:30:31 UT on 29 March 2014 (Figure 13 panel (f)). Figure 13 panel (g) shows a side view of the field lines in Figure 13 panel (f), where the final flux rope structure has approximately one turn along its length. Both the sign of helicity injection and chirality of the filament are of the dominant type found for the southern hemisphere.

To produce a magnetic structure that is consistent with the filament at the correct time and location, an additional form of injection of magnetic helicity is required. Through carrying out a number of additional simulations with a variety of rates, we find that the helicity density injection rate has to be between $3.75\,\mbox{--}\,5 \times 10^{16}~\mbox{Mx}^{2}\,\mbox{cm}^{-2}\,\mbox{s}^{-1}$ to reproduce the magnetic structure of the filament. It is important to point out that for the present simulations with open-top boundary conditions, where initially over 90% of the flux is open, the majority of this injected helicity is lost through the top boundary. The exact physical injection mechanism of this helicity at the present time is unknown. However, since flux emergence is an important part of the evolution of the active region over the time period considered, one possible scenario for its origin is the transport of magnetic twist from the interior of the Sun to the atmosphere. While we only show results for a single box size, start time, and initial condition, all three have been varied with and without additional helicity injection. In all cases, similar results are found since the main magnetic feature of interest always emerges after the initial condition is constructed, and as such, is treated the same regardless of the box size or initial condition used. While the present text gives a brief description of the results, a future study will consider this in more detail along with a full description of the quantities calculated.

From this modelling we determine the behaviour of the magnetic field configuration in the active region leading up to the X-class flare. The presence and qualities of the magnetic flux rope revealed along the PIL, considered in conjunction with our observational results, allows further conclusions to be drawn about the origin of the observed activity.

## Discussion

The pre-flare period of the 29 March 2014 X-class flare shows very dynamic phenomena occurring in multiple layers of the solar atmosphere. This activity is observed in both spectroscopic and imaging data.

Up to 40 minutes prior to flare onset, blue shifts are observed in multiple spectral emission lines by both IRIS and *Hinode*/EIS in two of the three subregions (Regions B and C) of study chosen. The first of these areas, Region B, is located on the filament seen in AIA and IRIS SJI data. Region B is also the site of non-thermal line widths of $70~\mbox{km}\,\mbox{s}^{-1}$, observed in the coronal Fe xii line. The plasma exhibiting this enhanced non-thermal velocity is also found to be blue shifted.

As detailed in Section 3.3 and shown in Figure 7, within this region we observe strongly blue-shifted Si iv, and He ii emissions of up to $200~\mbox{km}\,\mbox{s}^{-1}$. These emissions are also highly transient, with velocities peaking at 16:54 UT and 17:04 UT whilst falling to lower values at 17:00 UT. As earlier noted, this presents an interesting offset between the times of peak blue shift occurring in the lower atmosphere and the corona, where blue shifts are at a maximum at 17:00 UT. This offset could be caused, for example, by the propagation of plasma through the atmosphere or be the result of two separate but related phenomena *e.g.* small-scale reconnection at different heights in the atmosphere.

Region C also exhibits these strong transient blue shifts in Si iv and He ii data. As in the case of Region B, little activity is observed in these lines until ${\approx}\,16\mbox{:}45~\mbox{UT}$. After this time, $200~\mbox{km}\,\mbox{s}^{-1}$ blue shifts are observed until the onset of the X-flare. These blue shifts are very transient, particularly so in the Si iv line profiles, where they switch on and off during the 40 minutes prior to the flare at irregular intervals. This activity suggests that the process causing these flows is not constant. In both these regions the chromospheric response as determined by the Mg ii observations suggests that something is driving line broadening, particularly towards shorter wavelengths. However, as a result of the complex nature of the chromospheric lines, it is not a simple matter to say that these blue asymmetries are indicative of upflows. This effect is discussed in detail in Kuridze *et al.* (2015), who use simulations of $\mbox{H}\upalpha$ emission to show that changes in the optical depth of the solar atmosphere driven by upflows can lead to absorption of red-wing photons at a higher altitude, producing a blue asymmetry. This effect has also been noted by Kerr *et al.* (2016) to occur in Mg ii lines.

In Section 3.4 we detail the locations of the observed plasma flows. In particular, we highlight the IRIS Si iv observations where the transient blue-shifted flows (Figure 11) are found to coincide with an extended bright feature observed in AIA 193 Å data. These flows are fast, with blue-shifted velocities up to a maximum of ${\approx}\,200~\mbox{km}\,\mbox{s}^{-1}$, and with many regions exhibiting $100~\mbox{km}\,\mbox{s}^{-1}$ or upwards. These blue shifts and the related extended bright feature are very intriguing. The brightening and subsequent plasma velocities seen in both corona and transition region suggest that we are observing energy input into the region, possibly through reconnection. Testa *et al.* (2014) undertook a study of small brightenings at the foot-points of active region loops using IRIS data to investigate non-thermal particle heating produced by nano-flares. In these bright points, they identified blue-shifted plasma with typical centroid velocities of ${\approx}\,15~\mbox{km}\,\mbox{s}^{-1}$ and velocities of up to $40~\mbox{km}\,\mbox{s}^{-1}$ in the wings. These velocities are far slower than those that we have identified, leading us to conclude that nano-flare activity is likely not the driver of these flows and that we are not observing standard active region dynamics.

The non-potential magnetic field modelling described in Section 3.5 shows that a magnetic flux rope is present in the location of the filament over an hour prior to the X-class flare. The presence of a magnetic flux rope is also supported by Yang, Guo, and Ding (2016), whose modelling identified a flux rope in the active region from 09:00 UT. The inferred presence of a flux rope within the active region before the flare allows us to confine our ideas of what could be driving the observed flows. Strongly blue-shifted flows of up to $200~\mbox{km}\,\mbox{s}^{-1}$ in the transition region have been observed in pre-flare IRIS data by Cheng, Ding, and Fang (2015). In this article, the authors presented observations of two active regions prior to flare events. Within these areas studied, blue shifts of $200~\mbox{km}\,\mbox{s}^{-1}$ were identified to occur in the presence of filaments. The authors interpreted these fast upflows to be the result of magnetic reconnection occurring between two smaller flux ropes, to create a larger flux rope. The authors also identified downflows at the foot points of the final flux rope. These strong flows were observed in Ca ii, Mg ii, and Si iv. Cheng, Ding, and Fang (2015) did not investigate the coronal response to this activity, but comparing this work to our own observations, we note that the presence of strong flows located in the centre of the filament or flux rope, and observed from the chromosphere through to the corona, compares well. We do not observe the foot-points of the filament or flux rope with the spectrometers and so cannot comment on whether this piece of the Cheng, Ding, and Fang (2015) interpretation is present in our results. The presence of the flux rope in the region of study is another piece of evidence in favour of using the Cheng, Ding, and Fang (2015) model to interpret this pre-flare behaviour.

Kink instability is another possible mechanism through which solar flares can be triggered. If the kink instability were to be the driver for the flare we observe, we would expect to identify both red- and blue-shifted plasma along the flux rope (*e.g.*, Williams *et al.*, 2009). From our spectroscopic observations, we identify only blue shifts along the length of the filament. This casts significant doubt on the kink instability being responsible for the activity. The weakly twisted nature of the magnetic flux rope identified through our modelling also serves to further rule out the kink instability as the driver. We determine that the level of twist in the flux rope is insufficient to become kink unstable. We therefore conclude that the kink instability is not the driver of the observed activity due to the findings of our spectral observations and modelling.

Tether-cutting reconnection and magnetic breakout may also be considered as possible explanations for the strong flows and extended pre-flare brightenings observed. Activity associated with the breakout model would be expected to occur away from the filament. As the observed activity is seen to lie along the filament, we conclude that it is unlikely that reconnection due to magnetic breakout is the driver of this activity within the spectrometer fields of view. Tether-cutting reconnection proves to be a better candidate to explain this activity as related brightenings have been observed close to filaments (Warren and Warshall, 2001). The work of Kleint *et al.* (2015) showed the filament in this active region to be slowly rising in the hours before the eventual eruption, which would also be consistent with the tether-cutting model.

**Figure 14:**
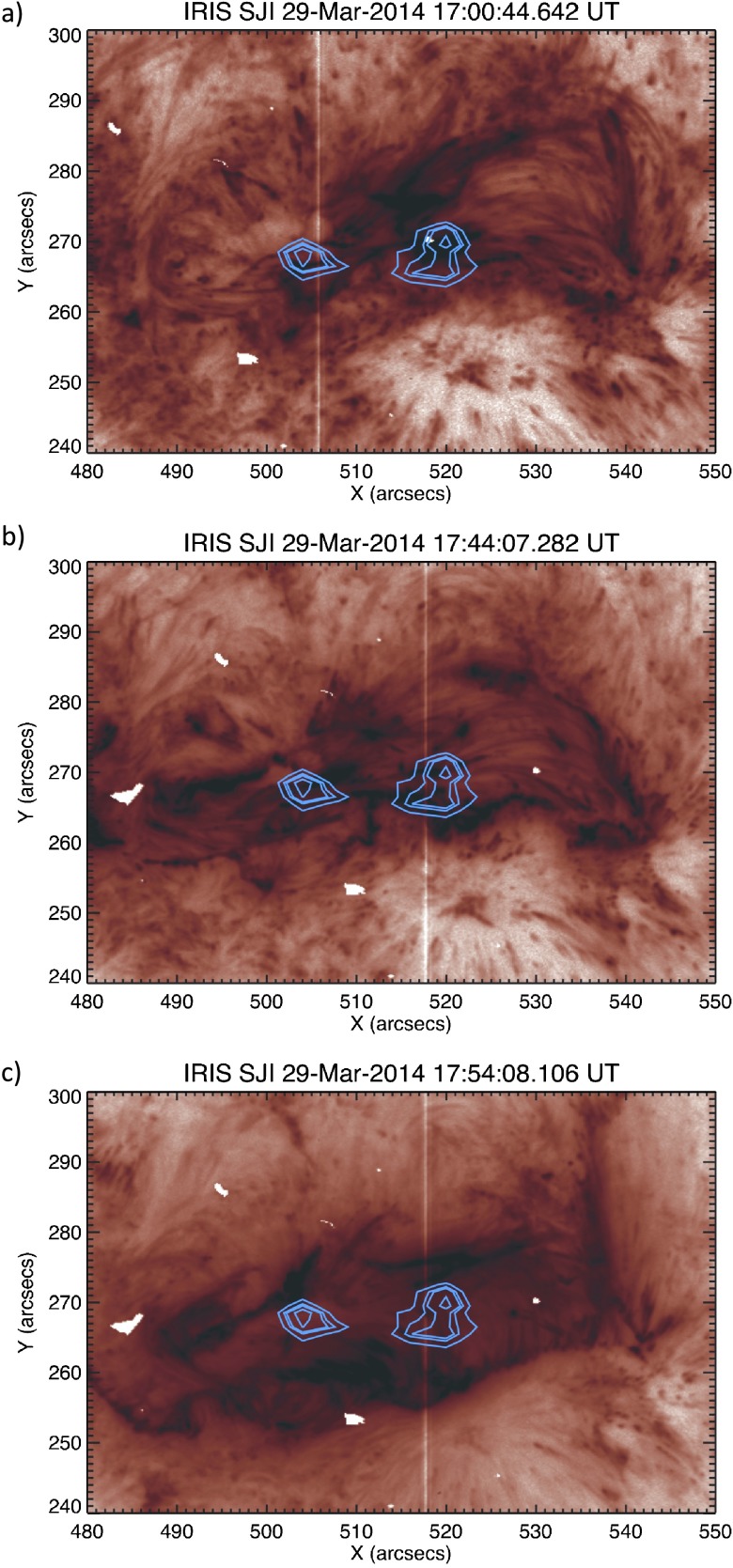
Comparison of the position of blue shifts observed in *Hinode*/EIS Fe xii data at $-100~\mbox{km}\,\mbox{s}^{-1}$ with flare ribbons identified in IRIS slit-jaw imager 1400 Å channel (an inverted colour table has been used). Panel (a) shows the position of the blue shifts at the time they occurred. These areas of blue shift are aligned with brightenings also visible in AIA 193 Å data. Panel (b) shows the blue shifts superimposed onto SJI data during flaring. This image is chosen as it is the closest image to the flare peak that is not saturated. Flare ribbons are visible and appear to lie close to the brightenings seen in earlier SJI and AIA 193 Å data. Panel (c) shows the situation post flare. We can clearly see the flare ribbons have expanded outwards from their initial positions.

As the extended bright features occur in the region of the flare and filament eruption, we investigate whether there could be a relation to the flare ribbons. In Figure 14 we show *Hinode*/EIS Fe xii data at $-100~\mbox{km}\,\mbox{s}^{-1}$, observed at 17:00 UT and overlaid on to IRIS slit jaw images in the 1400 Å pass band at three separate times. In panel (a) we see the extended brightenings at 17:00 UT that are discussed in Figure 11, linking the two regions of *Hinode*/EIS blue shift. During the flare at 17:44 UT (panel (b)), we can see the appearance of flare ribbons and their locations. There appear to be ribbon brightenings in close proximity to the locations of the *Hinode*/EIS blue-shifted regions. Strong brightenings are also clearly seen to lie along the path of the filament as it appeared prior to its eruption. The slit-jaw imager data saturate as the peak of the flare is reached. Panel (c) shows the situation at 17:54 UT, after saturation of the data has passed. Here we see that the flare ribbons are more obvious and have expanded outwards from their earlier positions. Flare ribbons are commonly observed during a flare, but have been identified prior to the impulsive phase. Fletcher *et al.* (2013) identified flare ribbons appearing to lie within a filament minutes prior to flaring, after which they underwent outward expansion. The locations of the extended brightenings in panel (a) and the flare ribbons in panel (b) are both very close to the location of the filament. This could suggest that the extended brightening may have a relation to the flare ribbons, but due to their appearance ${\approx}\,40~\mbox{minutes}$ before onset of flaring, it is highly doubtful that they are actually ribbons themselves.

**Figure 15:**
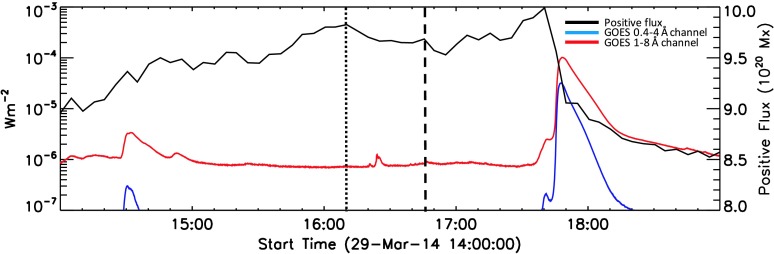
Evolution of positive flux with time over-plotted on the GOES light curve. We can see that over the period of joint EIS/IRIS observation, there is sustained flux emergence into the region of study. This emergence continues until 16:10 UT, highlighted by the dotted line. The nadir of this drop is coincident with the C-class flare at 16:24 UT. The flux then stays constant until 16:46 UT, where a second smaller drop in flux is observed (dashed line). This drop in magnetic flux is broadly coincident with the onset of the flows described in Section 3.2. This decrease in flux recovers quickly to its previous level. Again the flux remains roughly constant until 17:30 UT, where a large increase in the flux is observed prior to the steep drop coincident with the X-flare at 17:35 UT.

The work of Kusano *et al.* (2012) and Bamba *et al.* (2013) proposed and provided observational evidence for small-scale flux emergence at the PIL of an active region being related to the triggering of flares. For the active region studied in this article, Figure 15 shows the evolution of positive magnetic flux compared to GOES soft X-ray flux between 14:00 UT and 19:00 UT on 29 March 2014. We see a general trend of increasing magnetic flux over time. A gradual decrease in magnetic flux is observed from 16:10 UT, marked by the dotted line in Figure 15. The nadir of this decrease is coincident with the 16:24 UT C-flare. The flux level then plateaus for ${\approx}\,25~\mbox{minutes}$ until another drop is observed (marked by the dashed line in Figure 15). This second decrease coincides with the onset of the strong flows that we have observed throughout the solar atmosphere around 17:00 UT. The flux level increases once more to a maximum value of $9.99\times10^{20}~\mbox{Mx}$ just prior to the onset of the X-flare at 17:35 UT. At this time, a rapid decrease in magnetic flux is then observed in conjunction with the impulsive rise in GOES soft X-ray flux. This behaviour is consistent with the events designated as opposite polarity (OP) in Bamba *et al.* (2013), where small bipole fluxes that emerge along the PIL are of opposite polarity to the active region into which they emerge. In this scenario, magnetic flux must rise to a critical level and rapidly decrease when the flare is triggered by an instability in the system. The apparent decrease in magnetic flux at the time of the strong flows also indicates that there is some process occurring in the same region that is leading to the loss of flux. This could be due to flux cancellation, with reconnection driving the observed flows in the upper atmosphere and the subsequent submergence of the resulting small post-reconnection loops being the observed flux cancellation. Alternatively, the decrease in magnetic flux at the time of the observed pre-flare activity could be due to plasma flows in the atmosphere, or a heating process exerting a pressure upon the magnetic field. This could change the inclination of the magnetic field, resulting in the observed decrease in magnetic flux due to line-of-sight effects.

## Conclusions

In this work we carried out the first simultaneous spectroscopic study of pre-flare activity in the solar atmosphere from the corona to the chromosphere. Using observations from *Hinode*/EIS and IRIS, we have identified the presence of strongly blue-shifted plasma flows with velocities of up to $200~\mbox{km}\,\mbox{s}^{-1}$ in Si iv emissions from the transition region. These strongly blue-shifted flows also appear to be related to an extended bright feature observed to lie along the filament by SDO/AIA and IRIS SJI data. In our efforts to investigate these observed features, we undertook non-potential magnetic field modelling of the region detailed in Section 3.5. This revealed the presence of a weakly twisted flux rope over one hour prior to the flare and filament eruption. Section 4 detailed how these results relate to existing flare trigger models. The models that do not fit our observations are as follows. i) Kink instability: based on the weakly twisted nature of the flux rope and the absence of evidence of untwisting (red and blue shifts along the filament), we determine that it is highly unlikely that the activity is driven by the kink instability. ii) Breakout reconnection: within the spectrometer fields of view, all brightenings and flows are observed to occur close to or within the filament. This therefore rules out the likelihood of this activity being explained by breakout reconnection within the field of view available. However, as the field of view of both spectrometers is small, we cannot rule out breakout reconnection occurring remotely from the area we observe.

More probable explanations for these flows and brightenings are that they are related to the following phenomena. i) Reconnection in the flux rope: the strongly blue-shifted plasma that we are observing in the centre of the flux rope may be driven by a process similar to that described by Cheng, Ding, and Fang (2015). ii) Early onset of flare reconnection: the positions of the plasma flows and brightenings in relation to the flare ribbons were investigated. It was found that the positions of the brightenings can be related to the flare ribbons that appear during the flare. This would imply that we are observing the onset of flare reconnection 40 minutes prior to the main flare onset. iii) Tether-cutting reconnection: the position of the strong flows and brightenings along the filament suggests that the observed activity may be the result of tether-cutting reconnection.

The pre-flare environment is very complex, and not all activity related to the different trigger mechanisms is unique. The strong flows that we observe could be indicative of several possible flare drivers. We are therefore unable to provide conclusive evidence of one mechanism that drives the onset for the flare. Because of the complex nature of the solar atmosphere at the time the activity is observed, it is perhaps likely that some combination of all the possible trigger mechanisms drives the observed activity.
